# DNA fingerprinting using AFLP markers to search for markers associated with yield attributes in the silkworm, Bombyx mori

**DOI:** 10.1673/2006_06_15.1

**Published:** 2006-09-01

**Authors:** Duverney A. Gaviria, Enrique Aguilar, Herman J. Serrano, Alvaro H. Alegria

**Affiliations:** Center for Molecular Biology and Biotechnology, Universidad Tecnológica de Pereira, Risaralda, Colombia

## Abstract

This study was carried out on 11 Chinese and 12 Japanese silkworm strains maintained by the Center for the Technological Development of Sericulture (CDTS) germplasm bank, located in Pereira, Colombia. The goals were to determine the genetic population structure of the two groups and the association between molecular markers (AFLPs) and important productivity characters. Group analysis showed the separation of the strains according to their geographic origin. The molecular markers and the productivity characters were correlated by multiple variance analysis. The analysis permitted the identification of molecular markers associated with the cocoon weight or the shell weight separately. Some markers were associated with both characters.

## Introduction

Molecular markers possess several advantages over morphological characteristics, since they are not modified by the environment and can be identified at any developmental stage. Several techniques have been used in an attempt to identify molecular polymorphisms in the silkworm, including enzyme markers such as hemolymph amylases in tropical and temperate zones (Abraham *et al.* 1992;Goldsmith 1995). Using RAPDs Nagaraju *et al.* (1995), separated 13 silkworm strains in two groups, six with diapause and seven without diapause. Several additional groups markers were also identified. Using a probe (BKm –2, GATA_66_ TA) the two groups were clearly separated, and sex-specific bands were also identified. Damodar *et al.* (1999) analyzed 28 microsatellite loci (SSR) and identified a marker, sat 211, with specific alleles for strains with and without diapause. A very polymorphic marker, sat 2763, was also identified with a total of 17 alleles and very high heterozygozity (90%). These results could be very useful in the study of geographical relationships and in the analysis of wild strains or those in danger of extinction. Similar results with these same strains were obtained by Reddy *et al.* (1999), using other techniques.

Cocoon weight and shell weight are the main traits evaluated for productivity in sericulture and have been used for more than half a century. Cocoon weight is an important commercial characteristic used to determine approximately the amount of raw silk that can be obtained. Shell weight gives a better measure, but cannot be determined in commercial cultures because it requires damaging the cocoon. The difference between the two measures is the weight of the pupa. Using AFLPs the correlation found between these two parameters in this study was 70%. It was concluded that at least four genes affect shell weight and eight affect cocoon weight. One of the genes involved has been tentatively located in the sex chromosomes, both for cocoon weight and shell weight. However no definitive location has been reported so far (He *et al*, 1998).

A large number of morphobiochemical markers have been linked to molecular markers (Shi *et al.* 1995; Yasakuchi 1998; Nagaraju 2000). However, little has been done to understand the genetics of productivity traits, except in the analysis of heritability and combinatorial ability (Tazima 1964;Shibukawa *et al.* 1986; Rao *et al.* 1991; Chaterjee *et al.* 1993). DNA markers closely linked to a characteristic of interest could be used to select for that trait in different ways such as: 1) a segregating population of F_2_ crosses, using an analysis of bulked segregants (Michelmore *et al.*1991); 2) recombinant endogamic lines (Reiter *et al,* 1992); 3) near isogenic lines produced by improvement programs (Martin *et al.* 1991). However, within a intraspecific context, the quantitative trait loci mapping of many important agronomical characteristics require using very informative markers that allow the discrimination of a large number of bands (Tan *et al.* 2001). Of course, the affectivity of such marker-assisted selection depends on the precision of the phenotypic classification of the trait of interest and the degree of linkage between the marker and the trait (Nagaraju *et al.* 2002). The advances in statistical modeling and mathematics applied to genetic processes are also helping to optimize the use of the molecular and phenotypic information in selection programs (Bovenhuis *et al.* 1997; Hoeschele *et al.* 1997). When the characteristics of a population prevent the use of the mentioned techniques, alternatives such as statistical models including multiple regression analysis, or discriminating factor analysis, may be used to identify a few markers strongly linked to one or several productivity components. These can be later examined by progeny analysis and productivity pattern segregation, leading to the identification of the genes associated with this character in particular (Chaterjee *et al,* 2003).

The present study was carried first to determine the degree of diversity and the existing relationships between the different accessions belonging to the silkworm germplasm bank of the Center for the Technological Development of Sericulture (CDTS), using amplified fragment length polymorphisms (AFLPs) (Vos *et al.* 1995). Then, an effort to identify molecular markers associated with productivity characteristics such us cocoon and shell weight was attempted, through a statistical approach based on multiple variance and simple correlation analysis.

## Materials and Methods

### Insects

The characterization by molecular methods was carried out on 23 silkworm lines, 12 from Japanese (KOI, K02, KO5, K10, K20, K30, K40, K522, SG3, SG2, NG, KNA) and 11 from Chinese origin (CA, CBS, CC, CJ, CGS, CHS, CLS, CTS, SC1, SC2, SC3) that belong to the germplasm bank of the Center for the Technological Development of Sericulture (CDTS). They are kept in the farm “El Pílamo”, Pereira, Risaralda, Colombia.

For the linkage analysis between productivity traits and molecular markers, 160 cocoons were studied, distributed equally in Japanese lines (4 of high and 4 of low productivity), and Chinese lines (4 of high and 4 of low productivity). The productivity values were based on statistical data obtained at the Center for about ten years. Each line was separated into males and females and five replicas were carried out with each line to homogenize variance, for a total of ten individuals for each line (Table 1)

**Table 1.**
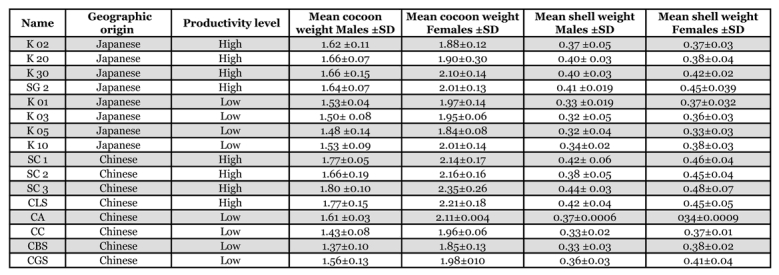
Productivity data

### Quantitative data

Cocoon weight was determined in each case. The weight of the shell was also measured by eliminating the pupa in each cocoon. An electronic balance was used and the data obtained to two significant figures.

### DNA extractions

DNA was obtained from two different tissues (silk gland and pupa) using a different protocol for each, Suzuki (1972) and Chatterjee (1993) respectively. In the extraction from the silk gland grinding of the tissue (0.04 g) was carried in 1 ml extraction buffer (NaCl 50mM, EDTA 50mM, SDS 1%), at 50°C with 10 μl of K-proteinase (100 mg/ml). For extraction of pupae, 1 ml of buffer was used (glucose 200 mM, EDTA 50 mM, Tris HCl 100 mM, SDS 0.5%, pH: 9.0) plus 2 μl K-proteinase (100 mg/ml) at 55°C. The tissue was incubated and ground periodically until there were no visible fragments; the samples were then centrifuged for 5 min at 14,000 rpm. 10 μl of potassium acetate (8M) were added to the extract before centrifugation. The aqueous phase was recovered and re-extracted with an equal volume of a chlorophorm- isoamyl alcohol mixture (24:1) for 5 min. After centrifugation at 14,000 rpm (Eppendorf 5417 R centrifuge) for 5 min. two volumes of absolute ethanol and 1/10 sodium acetate 3M were added to the separated aqueous phase to precipitate nucleic acids. After 5 min of centrifugation the supernatant was discarded and the pellet was air-dried. The pellet was re-suspended in TE buffer (Tris HCl 10mM, EDTA 1mM, ph: 8.0) and incubated one hour at 37°C after the addition of 10 μl pancreatic RNAse A (100 μg/ml). The DNA was re-extracted with phenol-chloroform-isoamyl alcohol and ethanol precipitated as before. The DNA was re-suspended in TE buffer, quantified by optical absorbance and examined by electrophoresis in an agarose gel (0.8%) stained with ethidium bromide. To further examine the DNA quality, samples were digested with the restriction endonuclease *Msp* I. When the sample did not digest properly, the DNA was submitted to a new round of extraction as indicated above.

### Primer selection for AFLPs

AFLPs reactions (Vos *et al.* 1995) were carried out using the “AFLP Analysis System I” kit from Invitrogene (Cat 10544-013), according to the manufacturer recommendations. Selective amplification by PCR was performed using a three nucleotide extension for both primers. To characterize the AFLPs four primer combinations were selected which gave a high number of non-ambiguous polymorphisms after 10 combinations were tried on four silkworm lines. For the identification of markers associated with productivity traits, five combinations were selected on a random basis.

PCR products were mixed with 10 μl loading mixture for sequencing (formamide 98%, EDTA 10 mM, xylencyanol 0.025%, bromophenol blue 0.025%), heated for 3 minutes at 90°C and then cooled on ice. 5 μl were then loaded on a 6% sequencing polyacrylamide gel and run at 90 W during 90 minutes. The gel was silver stained using the “Silver sequence^Tm^ DNA Sequencing System” kit from Promega (www.promega.com/), according to the manufacture recommendations. Permanent records were obtained by digital photography, transferring the images to TIFF format using a Nikon D’100 camera.

### Statistical Analysis

DNA bands were taken as a genetic locus, assuming that allele markers corresponding to different loci do not co-migrate and that each locus can be taken as a two allele system (Lynch and Milligan 1994). Data matrices were built for the presence (1) and the absence (0) in each of the loci. Zero (0) and one (1) matrices were obtained with the program for image analysis for molecular data “Gene Profiler ver 4.05” from Scanalytics, (www.scanalytics.com/).

### Similarity and group analysis

The determination of the relationships between the 23 silkworm lines was based on matrices built with the similarity indices of Nei and Li (1979). Dissimilarity values were analyzed using the Unweighted Pair Group Method with Arithmetic Mean (UPGMA) and a dendrogram was built. A principal coordinate analysis (Gower 1966) was also carried out to show the distribution in three dimensions. Both graphic representations made use of the statistical package NTSYS-pc, ver 2.01 (Rohlf 1992).

### Descriptive statistics and diversity

The homocedasticity assumption was revised using a Bartlett test (Sokal and Rohlf 1981) before each analysis was run. The programs POPGENE, ver 1.32 (Yeh *et al.* 1997) and TFPGA (Tools for Population Genetic Analysis), ver 1.3 (Miller 1999) were used to calculate, under the Hardy- Weinberg equilibrium assumption, the following parameters: 1) Recessive allele frequency and, accordingly, that of the dominant allele using the Taylor expansion method (Lynch and Milligan 1994); 2) Polymorphic loci percentage; 3) Non biased heterocigocity mean for all loci (Nei (1978)), neutrality test, and exact test of population differentiation (Raymond and Rousset 1995). These analyses were carried out both with the raw data and data to which an analysis restriction had been applied to avoid the bias in bands with a frequency higher than 1-(3/N) (Lynch and Milligan 1991). Data discussed in Results and Discussion refer to the corrected data.

### Population structure

The program AMOVA-PREP (Miller 1998) was used to transform the set of dominant data for the Molecular Analysis of Variance program (AMOVA, ver 1.55. Excofier *et al.* 1992). The data were analyzed as two populations and also as two subpopulations inside each population, in order to determine the part contributed by each one of the subdivisions to the total variance component. The analyses were carried out using both the raw data and data corrected with the factor 1- (3/N).

### Genetic distance

Genetic distance was determined for the populations and subpopulations using the TFPGA program and the calculation described by Nei 1978.

### Statistical analysis for the association of molecular markers and productivity traits

In order to determine which markers have a significant influence on productivity, multiple variance analyses were carried out for all samples. Shell weight first and then cocoon weights were considered as dependent variables and line, sex and marker as explanatory variables. Marker action on the character was determined by a lineal correlation analysis. All of these analyses were carried out using the Statgraphics plus, ver 5.1, software (www.statgraphics.com/). Shell and cocoon weight were selected as study variables since they are the main parameters to measure productivity. Each variance analysis was repeated, eliminating each time the explanatory variable with the less significant contribution until all the remaining variables made a significant contribution to productivity. This model succession allows detecting those factors with global importance, that is, those that are significant not only when compared with the dependent variable but also in the presence of other determining factors. These analyses were made for the markers grouped according to the primer combinations. Significance was set at 95%. The proposed method allows not only for the identification of groups of important factors, but is also theoretically more satisfactory than multiple regression analysis. The variance analysis works for discrete variables without violating assumptions or assuming normality, which would be wrong in this case (Sokal and Rohlf 1981).

The problem of variance uniformity was studied among the obtained markers, since the model does not fit this assumption strictly. In this respect, markers with a presence or absence less than 5%, were eliminated to eliminate false positives, and for the rest, variances were given according to which the significance of the marker in the variance analysis was determined. Using a regression and identifying which markers show a positive slope, those with a positive influence were defined. This regression implies only that the slope is positive or negative by least squares, without additional statistical assumptions.

## Results and Discussion

### Similarity and group analysis

To carry out the analysis, groups were defined from the dendrogram clusters. Accordingly, two principal groups were set up: Chinese and Japanese lines. From these, subgroups were tentatively defined, based again on the dendrogram clusters. The significance in the selection of the groups was statistically proved using the population differentiated exact proof (Raymond and Rousset 1995). The structuring level corresponding to subgroups was found non significant but the group level was significant.

**Figure 1.**
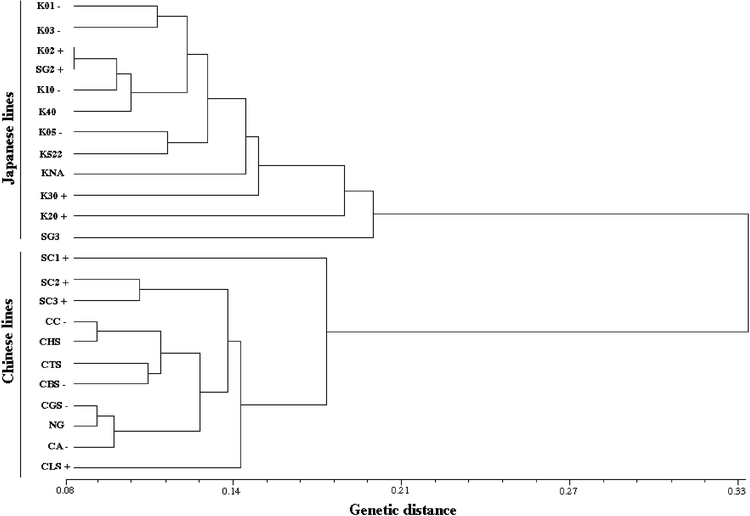
Dendrogram obtained by UPGMA indicating the relationships between the 23 CDTS silkworm lines. (168 AFLP bands). Signs (−) and (+) indicate lines with low or high productivity respectively.

**Figure 2.**
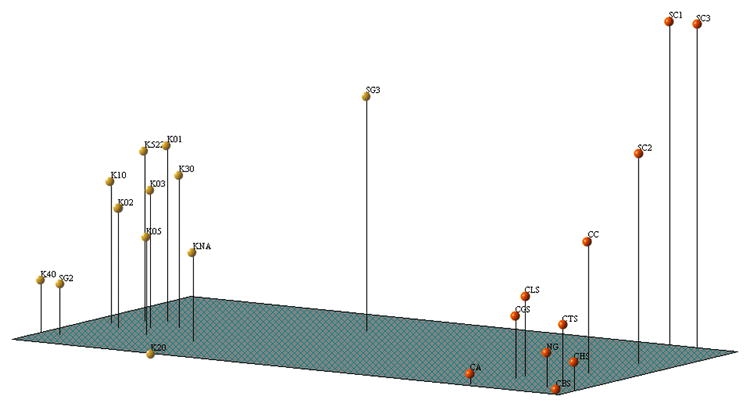
Principal coordinate analysis using a simple pairing matrix, for the data (168 AFLP bands). Yellow color are Japanese lines, red color are Chinese lines.

Individual analysis of each of the used combinations gave similar results and presented correlation indices for the data of the different matrices higher than 90%, indicating that the number of combinations used was sufficient for exploring the diversity present in the silkworm lines studied. Since the combinations gave highly concordant results, it is assumed that the whole genome was being analyzed (Barker *et al.* 1999; Rouppe *et al.* 1997; Zhu *et al.* 1998). The dendrograms corresponding to each of the primer combinations gave similar groupings (Figures 1 and 2).

The level of polymorphism for each combination was in the range 65.6–75.8%, although a large number of restriction fragments (247 bands) were generated in individual samples by AFLPs (Table 2). For analysis only those fragments that were polymorphic and registered without ambiguity were used. This correction was made according to the criterion established by Lynch and Milligan 1994, and finally a total of 168 bands were analyzed with an average polymorphism of 90.4% (Table 2). The rest of the analyses were carried out with the corrected data only.

**Table 2.**
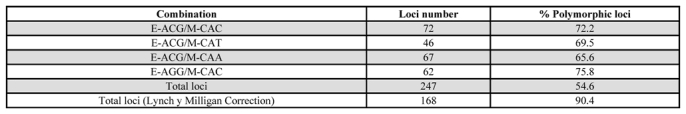
Used AFLPs combinations

The dendrogram generated by UPGMA analysis of the 168 bands (four primer combinations) resolved the 23 silkworm lines into two very well defined groups corresponding to the Chinese and Japanese lines (Fig. 1), with a distance of 0.22 for the Japanese lines and 0.18 for the Chinese lines. The analysis identified all the individuals.

### Descriptive statistics and diversity

For analysis, the following parameters were calculated for each combination: polymorphic loci percentage, heterocygocity, genetic flow, genetic distance, and Gst values (Fisher 1954). The data obtained using the four primer combinations show both in the dendrogram and in the principal coordinate analysis that the Japanese branch (distance 0.22) is more heterogeneous than the Chinese branch (distance 0.18) (Fig. 1 and 2). This observation was confirmed by the heterozygocity data, which were always higher for the Japanese lines. The estimate of the degree of population differentiation, given by the Gst value for each loci shows a high degree of differentiation (Table 3)

**Table 3.**
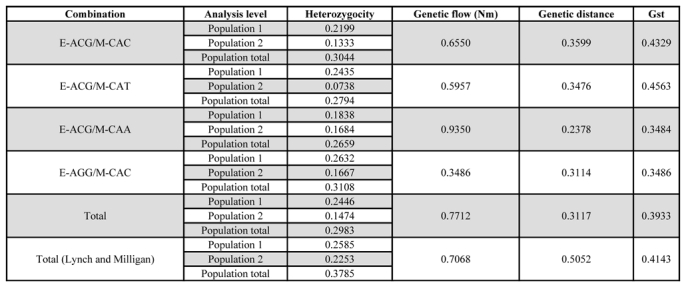
Descriptive statistical analysis for each primer combination and their sum.

### Population structure

The variance components were obtained by AMOVA (AMOVA, ver 1.55, Excofier *et al.*1992) using the corrected samples. Differences were analyzed between the evaluated races and inside each one. This analysis revealed that the variance components between populations were somewhat higher than inside each population, indicating some differentiation between the two races (Table 4). This result was further confirmed by a comparison of paired populations using Fst (Fisher 1954). Variance analysis was also carried out for the subpopulations defined by conglomerate analysis and group behavior was not observed, indicating that in these populations no subdivision levels with a lesser order exist (Table 5).

**Table 4.**
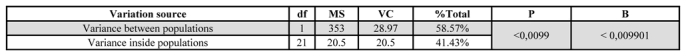
Corrected AMOVA: Variance component analysis between populations and inside each silkworm population.

**Table 5.**
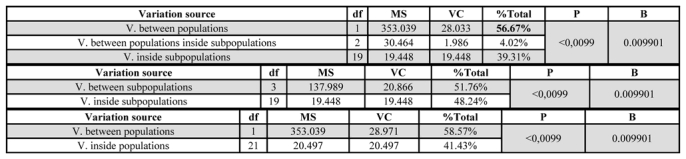
Corrected AMOVA: Variance component analysis between populations and subpopulations and inside populations and subpopulations.

### Association between AFLP markers and productivity characteristics

The number of markers generated from the analysis of 16 silkworm lines with five AFLP primer combinations was between 45 and 71, with an average of 60 AFLP markers distributed between 30–500 bp (Table 2).

To identify relationships (association, not linkage) between the DNA markers and the productivity characteristics, variance analysis for two characteristics against 302 molecular markers was made. Examples of the results are presented inTables 6 and 7. The analysis revealed the high degree of significance of sex and silkworm line in the explanation of productivity. The markers selected in each of the primer combinations for each of the productivity parameters are listed in Table 8.

**Table 6.**
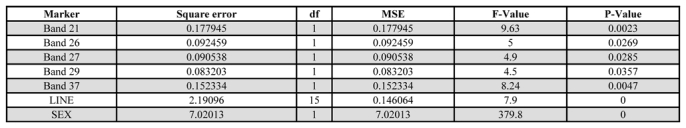
Variance analysis for cocoon weight/ square summation type III for the combination E-AAC M-CTA

**Table 7.**
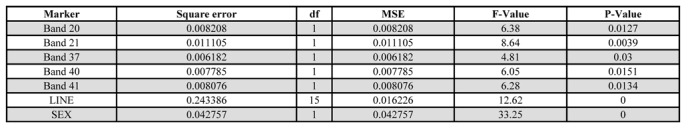
Variance analysis for cocoon shell weight/ square summation type III for the combination E-AAC M-CTA

**Table 8.**
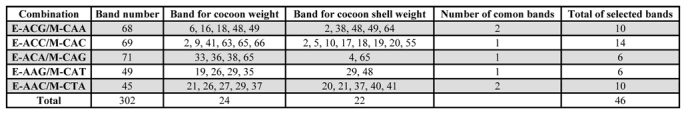
Identified markers for each of the evaluated characteristics

Marker identification was obtained through a process of successive elimination, one by one, of the markers giving non-significant results in the variance analysis. The number of markers used to carry out the selection varied from 2 to 8 for the combinations used for the evaluated characteristics. As seen in Table 8, common bands can be identified. In all 7 common bands were identified for the analyzed characteristics, one band for each primer combination on the average (eg. band 48 and 49 in the combination E-ACG/M-CAA) while others are specific for one of the characteristics only (eg. band 6 and 38 in the combination E-ACG/M-CAA). The type of contribution of each one of the markers for the evaluated traits was determined through a correlation analysis between each band and the particular trait. The number of selected markers for each individual primer combination is shown in Table 8. The number is variable and does not seem to have a direct relationship with the total number of bands for each combination. The total number of bands identified for each evaluated character was rather similar.

### Line genetic relationships

The present study shows that AFLPs can be used successfully in the silkworm to reveal DNA polymorphisms useful as genetic markers. The results demonstrate that this is a valuable technique for the discovery of genetic variation. In general, the information obtained by each primer combination seems to be the same. It can be concluded, therefore, that the analysis is covering the whole of the genome. The study allowed the identification of specific bands for each of the examined races, which can be very useful for identification of intellectual property rights and determining the purity of germoplams both for pure lines and commercial hybrids.

The conglomerates formed by this technique identified perfectly each of the lines according to their geographic origin, clearly indicating their Chinese or Japanese origin; this relationship is probably due to the process of endogamy by which the lines are maintained. In spite of the fact that the observed diversity was low (<10%), the results suggest that the lines that originated the Japanese race were originally less homogeneous than those for the Chinese race. In the CDTS collection, two Japanese lines were shown to be almost identical. Although more of the Chinese lines are similar, none showed this low degree of diversity. The geographic varieties of silkworm share a large genomic component and this degree of similarity makes it necessary to study and evaluate a higher number of characteristics in each line to determine if they should be maintained and replicated as a stock for improvement, in spite of their high degree of similarity. It would also be important to further evaluate lines such as SG3, K20 and K30 in the Japanese group and, above all, the SC1 Chinese line, which showed AFLP patterns that locate them distantly in their cluster.

The variance components suggest that it is necessary to broaden the genetic base, introducing new lines from the same or different geographic origin, if improvement programs are to be implemented to obtain higher productivity hybrids.

### Association between molecular markers and productivity components

The silkworm genome was found to be an excellent subject for analysis in fingerprinting programs for the identification of markers associated with productivity characteristics. Cocoon and shell weight are two very important economic traits that researchers have tried to physically identify in the genome for more than half a century. The present study tried to define markers present in a group of individuals with contrasting productivity characteristics without having an extreme distribution.

Different statistical approaches were applied to get an approximation to the association between molecular markers and productivity attributes. The fact that the information for each identifier (marker) is its presence or absence, prevent the use of regression inference because of the normality assumption. On the other hand, a multiple variance analysis does not make this requirement to its variables and allows conclusions about the significance of the contribution of each variable. The only point to discuss is the assumption of the uniformity of the variances. It can be argued, in this respect, that extreme cases in which the presence proportion was lower or higher than 5% were avoided and the method was used without evaluation of the individual variances. Multiple variance analysis allowed the identification of 46 marker bands, using a small number of AFLP primer combinations showing a significant association with the two productivity components studied.

Such markers might help in programs of marker-assisted selection. The success of such selection programs depends exclusively on the degree of linkage between the markers and relevant loci such as quantitative trait loci (QTLs). These require additional studies for specific markers selected from this analysis.

The analysis showed the importance of sex in this type of approximation. This characteristic becomes one that must be always evaluated, to avoid the identification of characteristics associated with sex and that can be falsely associated with productivity. Females are bigger and eat more than males. However these qualities do not result in higher productivity, since the additional energy is used to make eggs, not silk.

Correlation analysis between bands and productivity characteristics showed both positive and negative associations. In some cases markers for both cocoon weight and shell weight were observed. The contribution of these common markers was positive for both in some cases and positive for one and negative for the other in other cases. This result shows that a higher cocoon weight does not necessarily reflect a higher shell percentage. These characteristics seem to be controlled by different genes, confirming the evidence reported by He *et al.,* (1998).
